# Supplementary material for: Identification of photoperiod-induced specific miRNAs in the adrenal glands of Sunite sheep (Ovis aries)
Source: Front Vet Sci. 2022 Jul 22;9:888207. doi: 10.3389/fvets.2022.888207 (PMC9354845; doi:10.3389/fvets.2022.888207)
Supplement: Supplementary file 1 [file Data_Sheet_1.ZIP › Supplementary file/miRDeep2 file.zip!/miRDeep2 file/Know_Structure/SPLP42A2/oar-mir-381.pdf]

miRBase precursor : oar-mir-381
Total read count : 1670
oar-miR-381-5p read count : 0
oar-miR-381-3p read count : 1670
remaining reads : 0

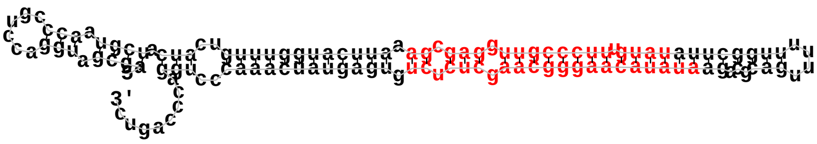

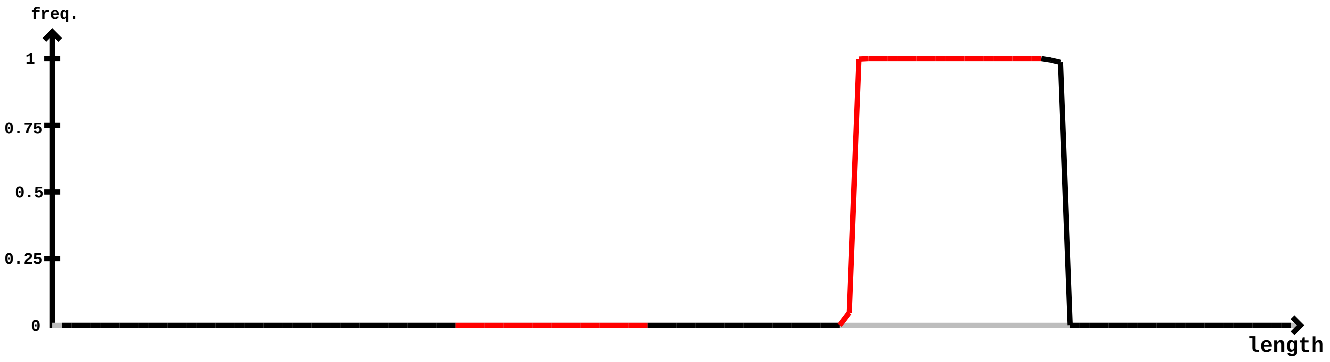


```
                                                                                 oar-miR-381-3p
                                         oar-miR-381-5p
5'- agcgauggaccugcccaaugcuacuacuguuugguacuuaaagcgagguugcccuuuguauauucgguuuuuugacgcagaauauacaagggcaagcucucugugaguaucaaacccuggacccaguc   -3'    exp
    ((((.(((......))).)))).(((..((((((((((((.((.(((.((((((((.((((((((((((....))).).)))))))))))))))))).))).)).))))))))))))..)))........    reads      mm
    .................................................................................auauacaagggcaagcucucug.........................    3          0
    .................................................................................auauacaagggcaagcucucugu........................    76         0
    ..................................................................................uauacaagggcaagcucucu..........................    9          0
    ..................................................................................uauacaagggcaagcucucug.........................    11         0
    ..................................................................................uauacaagggcaagcucucugu........................    1568       0
    ...................................................................................auacaagggcaagcucucugu........................    3          0
```
